# Extracellular vesicles and their effect on vascular haemodynamics: a systematic review

**DOI:** 10.1038/s41440-024-01659-x

**Published:** 2024-04-10

**Authors:** Sharon W. Y. Cheung, Lawrence W. Chamley, Carolyn J. Barrett, Sien Yee S. Lau

**Affiliations:** 1 Department of Obstetrics and Gynaecology, Faculty of Medical and Health Sciences, The University of Auckland, Auckland, New Zealand; 2 Hub for Extracellular Vesicle Investigations, The University of Auckland, Auckland, New Zealand; 3 Department of Physiology, Faculty of Medical and Health Sciences, The University of Auckland, Auckland, New Zealand

**Keywords:** Extracellular vesicles, haemodynamics, vascular reactivity, vascular tone, exosomes

## Abstract

Extracellular vesicles (EVs) are released from all cell types studied to date and act as intercellular communicators containing proteins, nucleic acids and lipid cargos. They have been shown to be involved in maintaining homoeostasis as well as playing a role in the development of pathology including hypertension and cardiovascular disease. It is estimated that there is 10^9^–10^10^ circulating EVs/mL in the plasma of healthy individuals derived from various sources. While the effect of EVs on vascular haemodynamic parameters will be dependent on the details of the model studied, we systematically searched and summarized current literature to find patterns in how exogenously injected EVs affected vascular haemodynamics. Under homoeostatic conditions, evidence from wire and pressure myography data demonstrate that injecting isolated EVs derived from cell types found in blood and blood vessels resulted in the impairment of vasodilation in blood vessels ex vivo. Impaired vasodilation was also observed in rodents receiving intravenous injections of human plasma EVs from cardiovascular diseases including valvular heart disease, acute coronary syndrome, myocardial infarction and end stage renal disease. When EVs were derived from models of metabolic syndromes, such as diabetes, these EVs enhanced vasoconstriction responses in blood vessels ex vivo. There were fewer publications that assessed the effect of EVs in anaesthetised or conscious animals to confirm whether effects on the vasculature observed in ex vivo studies translated into alterations in vascular haemodynamics in vivo. In the available conscious animal studies, the in vivo data did not always align with the ex vivo data. This highlights the importance of in vivo work to determine the effects of EVs on the integrative vascular haemodynamics.

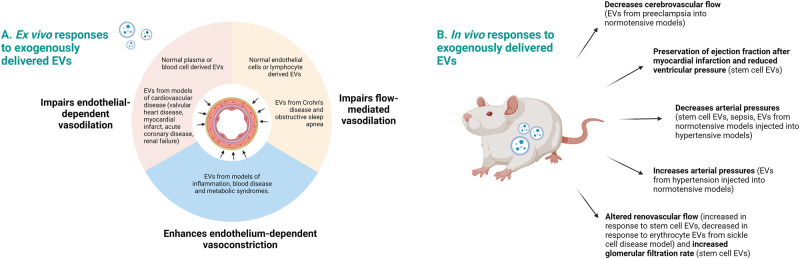

## Introduction

Extracellular vesicles are phospholipid membrane enclosed subcellular structures which can act as mediators of intercellular, inter-organ and inter-organism signalling [1]. This transdisciplinary field has grown exponentially within the last 20 years [1, 2]. Due to the literature on EVs growing exponentially we have a rapidly evolving understanding of EVs but with this comes a confusing proliferation of nomenclature [2, 3]. Most cells produce EVs that can be classified by their size as either large (>200 µm) or small (<200 µm) EVs [3]. Exosomes are subset of nano-EVs which are formed via a specific biosynthetic pathway in the multilamellar vesicles of the endosomes [3]. Unfortunately, many authors have used the term exosomes interchangeably with EVs in general but especially small -EVs. To avoid reinterpretation or misinterpretation of the literature, in this review we will use the terms used by the original authors to describe EVs. Similarly, there are several EVs isolation processes that are currently used by researchers which yield EV preparations which have various recovery rates and preparation purity. EV preparations, especially EVs preparations isolated from tissues such as blood can be contaminated with biomolecules including vasoactive substances that can at least in part contribute to any observed effects. It is difficult to ascertain in a review the purity of the EVs isolated in the included publications thus, we will assume that effects observed are due to the EV cargo and not contaminating impurities that were not removed in the isolation process.

It is well established that EVs carry bioactive cargoes that reflect the cell from which they originate. These cargoes can contain long and short non-coding RNA, mRNA, lipids, and proteins each of which may modulate the activity of recipient cells in both homoeostasis and pathology [4]. The large and growing interest in this field is also partly due to the potential therapeutic [5] and diagnostic roles of EVs. For example, increased levels of EVs have been found in preeclamptic (hypertensive) pregnancies compared to normal pregnancies [6, 7]. However, clarification regarding at which level of circulating EVs results in pathology and at what time point during gestation is needed before EVs can be used as a biomarker for the disease.

Hypertension was the cause of an estimated 10.4 million deaths globally in 2017 and the prevalence of this disease is on the rise due to the ageing population [8]. Hypertension is the largest risk factor for the development of cardiovascular disease (CVD) including stroke and ischaemic heart disease [9]. In hypertension, there is typically increases in systemic vascular resistance and altered cardiac output which combined, culminate in increased arterial pressure. The circulatory system carries EVs of various origins around the body enabling long-range communication between cells and can play a role in the altered physiology during hypertension [10]. While the dominant sources of circulating EVs are derived from circulating blood cells [11], there are EVs from non-cardiovascular sources such as hepatocyte-derived EVs [12] that can interact with and alter the function of the cardiovascular system.

It is estimated that there are approximately 10^10^ EVs present per mL of blood in healthy humans [13]. There is increasing evidence that the number and the nature of EVs found in the blood are altered in both cardiovascular disease [14] and hypertension [15]. For example, patients with stable coronary disease have increased numbers of circulating microvesicles [16]. In preeclampsia, a hypertensive disorder of pregnancy, it has been demonstrated that exogenous delivery of plasma EVs from patients with preeclampsia into mice impairs reactivity to vasoconstrictors in isolated murine arteries when assessed ex vivo using wire myography [17–19]. Given that EVs may potentially modulate haemodynamics, we conducted a systematic review to summarise the current literature on the effects of EVs from various origins on cardiovascular haemodynamics.

## Methods

### Literature search

A systematic search was performed on PubMed, Scopus, EMBASE and Web of Science databases up to 9th of September 2022. A search of the keywords (“extracellular vesicle”) AND (“total vascular resistance”) and to ensure coverage of the literature, synonyms for the keywords were included (Supplementary materials). Results were limited to original articles published in English.

### Eligibility criteria and search results

**Fig. 1:**
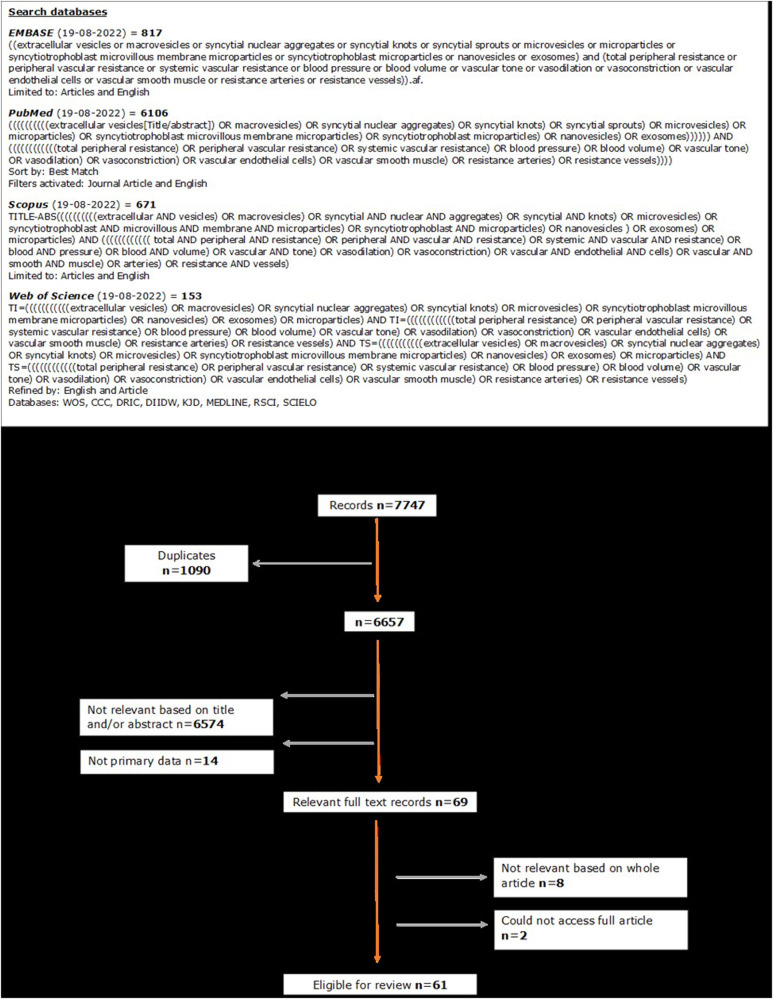
PRISMA flow diagram of systematic review literature search and data extraction process

A total of 7747 articles were retrieved from EMBASE (817), PubMed (6106), Scopus (671), and Web of Science (153). After removing duplicates, the remaining 6657 articles were screened, and 6574 articles were discarded as they were not relevant based on the title and/or abstract. A further 14 articles were discarded as they did not contain primary data, eight articles were discarded due to not being relevant which included two articles where we were unable to find a full text. This resulted in 61 full text records that were relevant to the review topic and eligible for review (Fig. 1).

### Data extraction

Relevant experimental results from each eligible article were compiled onto an Excel spreadsheet. The information of interest included type of EV, source of EV, method of EV isolation, experimental model, method of assessment, effect on vascular tone and arterial pressure.

## Results

### Ex vivo experiments used to investigate the effect of extracellular vesicles on vascular tone

We identified 33 of 61 articles which employed ex vivo techniques to investigate the effect of EVs on cardiovascular haemodynamics. These were performed using wire myography (37 studies, Table 1) or pressure myography (9 studies, Table 2) to assess the vascular reactivity to various vasodilators and vasoconstrictors following exposure to EVs. Wire myography measures the vascular reactivity by inserting wires into a vessel and attaching the wires onto an isometric force transducer. Contraction or relaxation in response to stimulation is detected by the change in force detected by the force transducer. In contrast, with pressure myography vessels are cannulated at both ends and changes in the vessel diameter are captured by a microscope and quantified. Pressure myography allows for assessment under static conditions (no flow through the vessel) in addition to dynamic flow, which induces intraluminal sheer stress and can be used to assess flow-mediated dilation, thereby more closely mimicking in vivo conditions [20].

**Table 1:** Summary of the effects of extracellular vesicles on vascular reactivity assessed by wire myography in the current literature

Conditions modelled/investigated	EV Type and Source	Recipient	Duration of EV exposure	Effect on constriction	Effect on dilation
Hepatocyte EVs	Rat small EVs [12]	Wistar rats [12]	Preincubated for 2 h [12]		Decreased acetylcholine-dependent vasodilation [12]
Placental EVs	Microvesicles and nanovesicles [35] Syncytiotrophoblast EVs [33] Nanovesicles [34]	Pregnant C57BL/6 and LOX-1 KO mice [35] Pregnant Sprague Dawley rats [33] Pregnant and non-pregnant CD1 mice [34]	Preincubated for 24 h [33, 35] 30 min or 24 h via intravenous injection [34]	Prolonged contraction to angiotensin II [35]	Decreased endothelium-dependent vasodilation • Methylcholine [33] • Acetylcholine (24 h) [34] Increased acetylcholine-mediated vasodilation (30 mins) [34] No effect on methylcholine-mediated vasodilation [35] No effect on sodium nitroprusside-mediated vasodilation [33–35]
Platelet-derived EVs	NZ White rabbit platelet microparticles [29]	NZ White rabbits [29]	Preincubated for 30 min [29]	Enhanced response to arachidonic acid and methacholine-mediated contractions [29]	
T lymphocyte derived EVs	Microparticles [24, 30, 40, 50] Human lymphoid CEM T cell line treated with actinomycin D or phytohemagglutinin microparticles [28]	Tissue engineered blood vessels [30] Swiss mice [28, 50]	Preincubated for 24 h [30] Preincubated for 4, 12 or 24 h [28] 24 h via intravenous injection [50]	No effect on contraction in response to histamine [30]	Decreased acetylcholine-mediated vasodilation [28] Increased acetylcholine-mediated vasodilation and restored endothelial function [50]
Endothelial EVs	HUVEC microparticles [23] C57BL6/J mice aorta endothelial cell microparticles [22] Rat renal microvascular endothelial cell microparticles [21]	BALB/c mice [23] Mouse model (unspecified) [22] Sprague-Dawley rats [21]	Preincubated for 24 h [23] Preincubated for 30 min [22] Preincubated for 3 h [21]		Decreased acetylcholine-mediated vasodilation [21–23]
Apoptotic MPs	Rat vascular smooth muscle cell microparticles [26] T lymphocytic cell microparticles [31]	C57BL/6 mice [26] Swiss mice [31]	Preincubated for 1 or 20 h [26] Preincubated or intravenous injection for 24 h [31]	Decreased vascular reactivity to serotonin [31] No effect on phenylephrine response [26]	Decreased acetylcholine-mediated vasodilation [26] No effect on sodium nitroprusside-mediated vasodilation [26]
Mesenchymal Stem Cell EVs	Human MSC-EVs [38]	Congenital diaphragmatic hernia model in new-born Sprague-Dawley rats [38]	Intravenous injection (circulation time unknown) [38]	Increased contraction response to endothelin-1 [38]	No effect on fasudil-mediated dilation [38]
Plasma EVs from inflammation • Non-muscular myosin-light chain kinase-deficiency (nmMLCK) • Crohn’s Disease	Mouse platelet-free plasma microvesicles [54] Crohn’s disease patients’ platelet-free plasma microparticles [78]	nmMLCK KO and C57BL/6 mice [54] Swiss mice [78]	24 h via intravenous injection [54, 78]		Prevented endothelial dysfunction and vascular hyporeactivity induced by lipopolysaccharide [54] Decreased acetylcholine-mediated vasodilation [78]
Myeloproliferative neoplasms	Microvesicles from platelet-free plasma of JAK2^V617F^ patients and JAK2^V617F^ mice [60]	JAK2^V617F^ and C57BL/6 mice [60]	Preincubated for 24 h [60]	Enhanced response to phenylephrine and angiotensin II [60]	
Cardiovascular disease	Normotensive and preeclamptic platelet-free plasma microparticles [17–19] Injured mouse placental-derived EVs [42] Wistar-Kyoto and Spontaneous hypertensive rat plasma exosomes [37] Human acute monocytic leukaemia cells transfected with miR-27a EVs [40] Normotensive and preeclamptic plasma microparticles [41] Myocardial infarcted and healthy patients’ platelet-free plasma microparticles [46] Valvular heart disease and healthy patient platelet-free plasma microparticles [48] Acute coronary syndrome patients platelet-free plasma microparticles [49] Myocardial infarcted and non-infarcted patients platelet-free plasma microparticles [47] End stage renal failure and healthy patients; platelet-free plasma microparticles [51] Human lymphoid CEM T cell line microparticles [39] Normotensive and preeclamptic plasma EVs [32]	Human pregnant omental arteries [17, 41] Human pregnant omental arteries and C57BL/6 mice [18] Pregnant C57BL/6 mice [19, 42, 48] Wistar-Kyoto and Spontaneous hypertensive rats [37] Sprague-Dawley rats [40, 47] Wistar rats [46, 51] Rat aortic rings (strain unspecified) [49] Angiotensin II induced hypertensive Swiss mice [39] C57BL/6 mice [32]	Preincubated for 24 h [17, 19, 46, 51] Preincubated for 24 to 48 h [32] Preincubated or intravenous injection for 24 h [18] Preincubated for 1 h [42] Intraperitoneal injections once a week (6 weeks total) [37] Intravenous injections twice a week (4 weeks total) [40] Preincubated for 1 h or overnight [41] Preincubated for 30 min [47–49] Intravenous injection three times in a week [39]	Preeclamptic MPs decreased vascular reactivity to phenylephrine and/or serotonin [17–19] Induced endothelial dysfunction and vasoconstriction [42] SHR exosomes increased serotonin-mediated contraction in SHR [37] WKY exosomes Reversed the increased prostaglandin F_2α_-induced contractions in SHR [37]	Decreased endothelial-dependent vasodilation • Angiotensin (1–7) [40] • Bradykinin [41] • Acetylcholine [32, 46–49, 51] Restored acetylcholine vasodilation [39] No effect on sodium nitroprusside-mediated vasodilation [32, 39, 46–49]
Metabolic syndromes • Diabetes • Metabolic Syndrome	Metabolic syndrome and healthy patients’ platelet-free plasma microparticles [55, 57] Diabetic patient plasma microparticles [31] Streptozotocin-induced diabetic rat PFP microparticles [58]	Swiss mice [31, 55, 57] Wistar rats [58]	24 h via intravenous injection [55, 57] Preincubated for 24 h [31, 58]	Decreased vascular reactivity to serotonin [31, 55]	Decreased acetylcholine-mediated vasodilation [57, 58] No effect on sodium nitroprusside-mediated vasodilation [58]
Cirrhosis	Cirrhosis and healthy patients’ platelet-free plasma microparticles [56]	Wistar rats [56]	Preincubated for 24 h [56]	Decreased vascular reactivity to phenylephrine [56]	
Obstructive sleep apnoea	Obstructive sleep apnoea and control patients’ platelet-free plasma microparticles [53, 59]	Swiss mice [53, 59]	24 h via intravenous injection [53, 59]	Increased vascular reactivity to serotonin and U46619 [59]	Decreased acetylcholine-mediated vasodilation [53] No effect on sodium nitroprusside-mediated vasodilation [53]

**Table 2:** Summary of the effects of extracellular vesicles on vascular reactivity assessed by pressure myography in the current literature

Source of EVs	EV type	Recipient	Duration of EV exposure	Effect on constriction	Effect on dilation
Endothelial cells	HUVEC microparticles [24] HMVEC EVs generated in the presence of ceramide or plasminogen activator inhibitor 1 [25]	C57BL/6 facialis arteries and human colon submucosal arterioles [24] Human adipose resistance arteries [25]	Incubated for 10 min [24] Preincubated intraluminally for 30 min [25]		Decreased acetylcholine-mediated vasodilation [24] Decreased flow-induced dilation [25]
T lymphocyte derived	Human lymphoid CEM T cell line treated with actinomycin D or phytohemagglutinin microparticles [28]	Swiss mice [28]	Not stated [28]		Decreased flow-induced dilation [28]
Sickle cell disease	Transgenic SAD (model for sickle cell disease) and wild-type mice erythrocyte microparticles [27]	Transgenic SAD mice and wild type mice [27]	Perfused intraluminally at time of testing [27]		Decreased acetylcholine and sodium nitroprusside-mediated vasodilation [27]
Hypertensive disease • Preeclampsia	Normotensive and preeclamptic patient, and Wistar-Kyoto and SHR platelet-free plasma EVs [10] Human lymphoid CEM T cell line microparticles [39] Normotensive and preeclamptic platelet-free plasma microparticles [18]	Wistar-Kyoto and Spontaneous hypertensive rats, and C57BL/6 mice [10] Swiss mice [39] C57BL/6 mice [18]	Added to organ bath and intraluminally at time of testing [10] Intravenous injection three times in a week [39] Preincubated for 24 h [18]		Normotensive rat EVs decreased acetylcholine-mediated vasodilation in normotensive vessels [10] Corrected angiotensin II induced impairment of flow-induced relaxation [39] No effect on flow-induced relaxation [18]
Obstructive sleep apnoea	Obstructive sleep apnoea and control patients’ platelet-free plasma microparticles [53]	Swiss mice [53]	24 h via IV injection [53]		Decreased flow-induced dilation [53]
Crohn’s Disease	Crohn’s disease patients’ platelet-free plasma microparticles [78]	Swiss mice [78]	24 h via IV injection [78]		Decreased flow-induced dilation [78]

#### Ex vivo assessment of the role of EVs in homoeostatic (non-pathological) conditions

The direct effects of endothelial cell-derived microparticles (earlier terminology used for large-EVs) under homoeostatic (non-pathological) conditions were investigated in five studies. These studies used primary cultured endothelial cells isolated in house [21–24] or purchased commercially [25] as the source of the EVs. Four studies reported impairment of endothelium-dependent acetylcholine-mediated vasodilation despite each study using a different animal model [21–23] or human arteries [24]. One study further demonstrated that while endothelial EVs impaired acetylcholine-mediated relaxation, in a model of oxidative stress, endothelial cell EVs prevented lipid-induced endothelial damage and restored endothelium-dependent vasodilation in mice aortas [23]. In one study, human resistance arteries were exposed to human cardiac microvascular endothelial cell (HMVEC) EVs generated following treatment with ceramide. Treatment with these HMVEC EVs for 30 min resulted in impaired flow-induced dilation in human resistance arteries after exposure [25]. These five studies suggest that EVs derived from endothelial cells restrain the endothelium-dependent vasodilation in response to acetylcholine, as well as impairing intrinsic flow-mediated vasodilation.

Similarly, apoptotic smooth muscle cell-derived EVs were shown to elicit a dose-dependent reduction in acetylcholine-induced relaxation in murine aortas but not in response to the non-endothelium dependent vasodilator, sodium nitroprusside [26].

Blood cell derived EVs were studied in five publications with one investigating erythrocyte derived EVs, one study investigating platelet derived EVs and three studies investigating EVs derived from lymphocytes [27–31]. Erythrocyte derived microparticles from wild-type mice impaired both endothelium-dependent (acetylcholine) and endothelium-independent (sodium nitroprusside) vasodilation in murine mesenteric arteries [27]. Exposure to platelet microparticles from NZ white rabbits was shown in one study to enhance the arachidonic acid-induced (endothelium-dependent) contraction in rabbit aortas and methacholine (endothelium-dependent) contractions in rabbit pulmonary arteries [29]. Both in vivo and ex vivo exposure to EVs from apoptotic T cells impaired contractile response to serotonin and phenylephrine in murine aortas with or without intact endothelium [31], whilst another study found exposure to human lymphoid CEM T cell-derived EVs (a lymphoblastic cell line) to impair flow-induced vasodilation in murine mesenteric arteries [28]. One study investigated the effect of exposure to hepatocyte-derived EVs, which were found to impair acetylcholine-induced relaxation in rat pulmonary arteries [12].

Four studies investigated the effects of placenta derived EVs on vascular tone [32–35]. During pregnancy, it is estimated that roughly 10% of all circulating EVs are derived from the placenta [36]. Murugesan and colleagues used wire myography to demonstrate plasma-EVs from severe preeclamptic pregnancies significantly reduced the response to endothelium-dependent vasodilation when the aortas were co-incubated with EVs for 24–48 h [32]. However, they did not observe a significant difference in endothelium-independent vasodilation [32]. These findings suggest EVs from the severe preeclamptic pregnancies impair endothelium-dependent vasodilation due to the downregulation of endothelial nitric oxide synthase. The following studies found that the effect of placental EVs on vasodilators appears to be time dependent. A longer exposure time (overnight or 24 h) resulted in impairment of endothelium-dependent relaxation, regardless of the gestation of the placenta [34, 35]. However, after 30 min exposure to nanovesicles (terminology used to reference small-EVs), Tong and colleagues also demonstrated an increased response to endothelium-dependent relaxation [34]. In a different study, human term placental EVs isolated from a perfused normotensive placenta demonstrated prolonged vasoconstriction in response to angiotensin II in pregnant wild-type murine uterine arteries [33]. In contrast, Tong and colleagues demonstrated human first-trimester placental EVs had no effect of phenylephrine and U46619 induced constriction in uterine or mesenteric arteries in both pregnant or non-pregnant mice [34]. These studies sourced EVs from placentae of different gestational ages, Spaans and colleagues used term placental EVs, whilst Tong and colleagues used first-trimester placental EVs. As the anatomy and function of the placenta changes dramatically over gestation, it is not clear whether EVs derived from these early and later gestations would be expected to give similar results. The effects of placental EVs on the response to vasoconstrictors and vasodilators in the current literature is yet to reach consensus due in part to the limited number of studies, different methods of generating placental EVs and/or isolation, as well as likely differences in the EVs produced from the placenta across gestation.

### Ex vivo studies assessing the role of EVs in hypertensive diseases

All studies discussed in this section used a model for arterial hypertension. Two studies investigated the effect of plasma-derived EVs from SHRs to model hypertension [10, 37]. The first study demonstrated that plasma exosomes derived from the SHR model increase the vasoconstrictor response to serotonin (endothelium-independent) and prostaglandin F2-α (endothelium-dependent) in vessels from SHRs [37]. However, when the SHR vessels were first exposed to plasma exosomes from SHRs, then exposed to plasma exosomes from Wistar-Kyoto controls, the increased contractile response to prostaglandin F2-α was supressed suggesting that Wistar-Kyoto rat plasma-derived exosomes corrected the SHR EV-mediated increase in contraction [37]. Another study investigated platelet-free plasma EVs from wild type and SHRs added to the organ baths and intra-luminally then tested the acetylcholine (endothelium-dependent) mediated vasodilation [10]. EVs from wild type rat reduced vasodilation in wild type rat arteries but not in SHR arteries [10]. However, SHR plasma-derived EVs did not impair vasodilation of either normotensive or hypertensive rat arteries [10]. This study highlighted that the response to EVs in models of hypertension depends on both the cargo of the EVs, as well as the phenotype of the arteries studied. In a separate experiment, Good and colleagues also used human platelet-free plasma EVs and found that EVs from normotensive human patients, but not EVs from hypertensive patients, impaired vasodilation of normotensive murine arteries [10]. This finding was consistent with their observation in rats. Taken together, it appears that plasma EVs from the hypertensive state do not impair endothelium-dependent vasodilation observed when exogenously delivering plasma EVs from in normotensives states but may exert a hypertensive effect by increasing the contractile response to vasoconstrictors.

Congenital diaphragmatic hernia (CDH) is a disease commonly associated with abnormal pulmonary vasculature, often resulting in pulmonary hypertension. The effects of human MSC-EVs on pulmonary artery vascular reactivity was assessed in a rat model for CDH [38]. After intravenous administration of MSC-EVs in new-born rats, endothelin-1 mediated vasoconstriction was observed to improve when compared to the untreated group [38]. In contrast, the endothelium-dependent vasodilation in response to fasudil was not affected [38]. The findings from these models of hypertensive states suggest vascular reactivity is dependent on the condition of the recipient, as well as the origin of the EVs.

A total of two studies used immune cell line-derived and kidney cell line-derived EVs to correct changes induced in arteries during the hypertensive state [39, 40]. One study used an angiotensin II-induced hypertensive mouse model to study the effects of CEM T-cell line derived microparticles [39]. Angiotensin II-induced hypertension model displayed impaired flow-mediated dilation in mesenteric arteries, which appeared to be corrected by the CEM T cell line derived microparticles [39]. Two studies used modified EVs to study hypertension. Activated human lymphoid CEM (lymphoblastic cell line) T cells were manipulated to generate sonic hedgehog carrying EVs, which were demonstrated to correct the impaired acetylcholine-induced relaxation in an angiotensin II infused murine model [39]. A subsequent study used miR-27a (which potentially targets expression of the Mas receptor gene) transfected THP-1 cells (monocyte cell line) and HEK-293 cells (kidney cell line) to generate EVs as a model of hypertension [40]. The response to angiotensin (1–7) mediated vasodilation was impaired in rat mesenteric arteries following exposure to the MiR-27a containing THP-1 and HEK-293 cell-derived EVs, and vasodilation was further impaired when exposed to EVs from transfected THP-1 cells that were lipopolysaccharide-treated [40].

The effect of platelet-free plasma (PFP) derived microparticles from women affected by preeclampsia, a hypertensive disorder of pregnancy, were investigated in four studies [17–19, 41]. Three of these studies demonstrated an reduced response to vasoconstrictors (serotonin and phenylephrine) in human omental and murine mesenteric arteries [17–19], while one study demonstrated abolition of the bradykinin-mediated vasodilation in uterine myometrial arteries when exposed the microparticles for 24 h [41]. Furthermore, the group found no effect of preeclamptic plasma EVs on bradykinin-induced (endothelium-dependent) relaxation when vessels were exposed to EVs for one hour [41]. When flow mediated vasodilation was assessed following 24 h preincubation of platelet-free plasma EVs from normotensive pregnant or preeclamptic women, no effect was seen in flow-mediated dilation in murine mesenteric arteries [18].

In an attempt to model the role of placental EVs in preeclampsia, one study obtained placentae from normotensive pregnant mice, which were snap-frozen with liquid nitrogen before being thawed and homogenised to collect vesicles via differential centrifugation [42]. These freeze-thawed placental-derived EVs induced vasoconstriction in murine aortas when the EVs were introduced in the organ baths [42]. A caveat of this model is the isolation of the placenta-derived EVs, as it is uncertain whether the freeze-thaw method of generating injured placental EVs is representative of preeclamptic EVs. Gupta and colleagues have previously shown that the function of EVs differs depending on the method used to generate them [43], while it has been previously demonstrated that freeze-thawed placental cell lines also produced relative increases in blood pressure in pregnant rats [44].

Taking together the findings from studies investigating the effects of EVs from hypertensive conditions, it appears that EVs isolated from pregnant hypertensive conditions impair contractile responses in the pregnant state when challenged with vasoconstrictors. In comparison, hypertensive EVs from a non-pregnant state (SHR) appear to enhance the constrictive response to vasoactive drugs. The discrepancy in the effects of EVs may be due to additional compensatory mechanisms in the pregnant state, as pregnancy favours a more pro-dilatory state [45]. The effects of EVs from a hypertensive model on vasodilation appear to be dependent on the model employed, whether that is cargo the EVs are carrying (sonic hedgehog) or the parent cell the vesicles were extruded from (miR-27a expressing cells), or the clinical presentation from whom the EVs were initially derived from (e.g., preeclamptic patients).

### Ex vivo studies assessing the role of EVs in cardiorenal pathologies

In different cardiovascular pathologies studied—myocardial infarct, acute coronary syndrome and valvular heart disease, plasma derived EVs appear to impair endothelium-dependent but not endothelium-independent vasodilation.

Four out of the five studies studying different cardiomyopathies found EVs to impair endothelium-dependent relaxation [46–49]. Isolated platelet-free plasma EVs from acute myocardial infarct patients impaired acetylcholine-mediated relaxation, and this effect was abolished after removal of the rat aorta endothelium suggesting a mechanism originating from endothelial cells [46, 47]. This effect was enhanced by EVs isolated from patients after a percutaneous coronary intervention surgery for the myocardial infarction [47]. Furthermore, the studies observed no effect of myocardial infarct-derived-EVs on endothelium-independent relaxation (sodium nitroprusside) [46, 47]. In a model of ischaemia and reperfusion of the heart, coronary arteries were demonstrated to have decreased relaxation to acetylcholine [50]. However, administration of human lymphoid CEM T cell-derived EVs prior to the ischaemic event completely reversed the impairment caused by the insult and enhanced the acetylcholine-mediated vasodilation [50]. Platelet-free plasma EVs from patients with valvular heart disease or acute coronary syndrome were also demonstrated to impair endothelium-dependent but not endothelium-independent vasodilation in mice [48] and rat aortas respectively [49].

One study investigating platelet-free plasma EVs from end-stage renal failure patients demonstrated an impaired acetylcholine-mediated relaxation response in rat aortas after incubation with EVs for 24 h [51].

### Ex vivo studies assessing the role of EVs in inflammation, metabolic and blood diseases

The following studies investigated EVs from pathological states in which the inflammatory process and vascular endothelium is activated, including sepsis, metabolic syndrome, cirrhosis, obstructive sleep apnoea (OSA), and diabetes.

Platelet-free plasma (PFP)-derived EVs from septic patients were demonstrated to increase the sensitivity to the vasoconstrictor serotonin in murine aortas from control and lipopolysaccharide-treated mice [52]. PFP-derived EVs from patients with Crohn’s disease, an inflammatory condition of the bowel, were demonstrated to impair both acetylcholine-mediated endothelium-dependent vasodilation and flow-mediated vasodilation in the mesenteric arteries after intravenous injection in mice for 24 h [53]. One study evaluated the platelet-free plasma EVs from a nmMLCK (non-muscular myosin light chain kinase) knockout mouse model prevention of endothelial dysfunction and impaired endothelium-dependent vasodilation and endothelium-independent vasoconstriction induced by lipopolysaccharide [54]. These workers found that EVs from wild-type mice were able to partially prevent the impaired vasoconstriction and vasodilatory responses while EVs from nmMLCK knock out mice had a larger effect [54].

Studies investigating PFP-derived EVs from patients with metabolic syndrome or cirrhosis demonstrated an impaired response to the contractile substances phenylephrine, serotonin, or U46619 in rat and mouse aortas [55, 56]. Exposure to PFP-derived EVs from diabetic patients for 24 h also impaired the contractile response to serotonin and phenylephrine in murine aortas with or without intact endothelium [31]. PFP-derived EVs from both patients with either metabolic syndrome or diabetes were also shown to impair acetylcholine-mediated endothelium-dependent vasodilation in murine aortic rings [53, 57]. One of the studies investigated the effects of platelet-free plasma EVs from streptozotocin-induced diabetic rats and showed no effect of these EVs on the vessel’s ability to dilate in response to sodium nitroprusside [58].

One study demonstrated that exposure to PFP EVs from OSA patients induced an increased constriction response to serotonin, U46619, and potassium chloride in murine aortas [51]. Additionally, the authors were able to demonstrate that the endothelium played a central role in this response as denuding the endothelium attenuated the enhanced response to the vasoconstrictors [59]. In a separate study, platelet-free plasma EVs from patients with OSA also impaired acetylcholine-mediated endothelium-dependent dilation and flow-induced dilation in murine mesenteric arteries 24 h after intravenous injection [53].

Erythrocyte-derived EVs from a JAK2^V617F^ murine model of myeloproliferative neoplasms caused an increased response to phenylephrine in wild-type mouse aortas [60]. This effect was not induced by platelet, peripheral blood mononuclear cell, or polymorphonuclear cells microvesicles [60]. In the same study, plasma microvesicles from patients carrying JAK2^V617F^ also increased the response to phenylephrine in the aortas of wild-type mice compared to microvesicles derived from control patients [60]. In the SAD mouse model of sickle disease, acetylcholine-mediated endothelium-dependent vasodilation was almost completely abolished after exposure to erythrocyte derived microparticles [27]. The authors also found the SAD erythrocyte microparticles to impair sodium nitroprusside-mediated vasodilation [27].

In inflammatory conditions (sepsis, OSA and Crohn’s disease), endothelium-independent vasoconstriction was enhanced whilst in Crohn’s disease and OSA, endothelium-dependent and flow-mediated vasodilation was impaired. This contrasts with metabolic conditions (metabolic syndrome and diabetes), where endothelium-independent vasoconstriction was impaired.

### In vivo experiments used to investigate the effect of extracellular vesicles on vascular haemodynamics

Studies investigating the effect of EVs on vascular dynamics in both conscious and anaesthetised animals is summarised in Table 3.

**Table 3:** Summary of the effects of extracellular vesicles on vascular haemodynamics assessed by in vivo methods current literature

Condition modelled/investigated	EV type	Recipient	Duration of exposure	Cardiovascular variable measured	Increased	No effect	Decreased
Plasma EVs from inflammation	Crohn’s disease patients’ platelet-free plasma microparticles [78] Septic, sham and healthy rat platelet-free plasma microparticles [71]	Conscious Swiss mice [78] Anaesthetised Wistar rats [71]	24 h via intravenous injection [78] Intravenous infusion at the beginning of procedure (7 h) [71]	Arterial pressure [71, 78]		No effect on systolic [78]	Decreased mean [71]
Sickle cell disease	Transgenic SAD (model for sickle cell disease) and wild-type mice erythrocyte microparticles [27]	Anaesthetised transgenic SAD and C57BL/6 mice [27]	Intravenous injection at beginning of assessment [27]	Peripheral arterial pressure [27]		No effect on arterial pressure [27]	Formation of vaso-occlusions [27]
Myeloproliferative neoplasms	Microvesicles from platelet-free plasma of JAK2^V617F^ patients and JAK2^V617F^ mice [60]	Anaesthetised JAK2^V617F^ knock-in and C57BL/6 mice [60]	Intravenous injection 2 h prior to experiments [60]	Peripheral arterial pressure [60]	Increased vascular reactivity to phenylephrine and angiotensin II [60]		
Cardiovascular disease • Hypertension • Preeclampsia • Hypoplastic Left Heart Syndrome	Small EVs from extravillous trophoblast cell line (HTR-8/SVneo) [77] Injured mouse placental-derived EVs [42] Rat bone marrow mesenchymal stem cell-derived microvesicles [63] Adipose mesenchymal stem cell-derived EVs [76] Wistar-Kyoto and Spontaneous hypertensive rat plasma exosomes [37] Hypo-tonically stimulated HEK293T derived exosomes [72] Rat adventitial fibroblast-derived exosomes [74] Human acute monocytic leukaemia cells transfected with miR-27a EVs [40] Human lymphoid CEM T cell line microparticles [39] Human normotensive pregnant plasma [75]	Conscious pregnant Sprague-Dawley rats [77] Conscious C57BL/6 mice [42] Anaesthetised Sprague-Dawley rats with pulmonary hypertension [63] Conscious Wistar-Kyoto and Spontaneous hypertensive rats [37, 74] Anaesthetised angiotensin II type 1 receptor knockout and C57BL/6 mice [72] Conscious Wistar-Kyoto DOCA-salt hypertensive rats [76] Conscious Sprague-Dawley rats [40] Conscious Swiss mice [39] Conscious Sprague-Dawley rat model for preeclampsia [75]	Intravenous injection once a day for four days [77] Intravenous infusion at time of assessment [42] Intravenous injection every two days for two weeks [63] Intraperitoneal injection once a week (6 weeks total) [37] 24 h via intravenous injection [72] Intravenous injection every two days over 20 days [74] Injections twice a week (4 weeks total) [40] Intravenous injection three times in a week [39] Single tail-vein injection [75]	Peripheral arterial pressure [37, 39, 40, 42, 63, 72, 74–77]	Increased mean [42, 77] Increased systolic [37, 40, 72, 74]	No significant improvement in blood pressure [75]	Decreased mean [63] Decreased systolic [37, 39, 74–76]
	Rat bone marrow mesenchymal stem cell-derived microvesicles [63] Human mesenchymal stem cell-derived EVs [65, 66]	Anaesthetised Sprague-Dawley rats with pulmonary hypertension [63, 65, 66]	Intravenous injection every two days for two weeks [63] One intravenous injection daily for up to five days depending on protocol [65] One intravenous injection daily for three days [66] Intravenous injection once a week for five weeks [66] Intravenous injection once every fortnight for 10 weeks [66]	Right ventricular pressure [63]		No significant increase from controls	Decreased mean [63, 65, 66]
	Hypoxic placenta-tissue derived EVs [42]	Anaesthetised C57BL/6 mice [42]	Intravenous infusion at time of assessment [42]	Cerebral blood flow [42]			Decreased [42]
	Bone marrow mesenchymal stem cell-derived microvesicles [64] Plasma and endothelial cell culture media with overexpression of miR-211 [68]	Anaesthetised Sprague-Dawley rats with pulmonary hypertension [64] Hypoxia-induced pulmonary hypertension Sprague-Dawley rats and normoxic controls [68]	Intravenous injection once every two days for 35 days [64] Tail vein injection [68]	Pulmonary artery pressure and pulmonary vascular resistance [64, 68]	Increased mean pulmonary artery pressure and pulmonary vascular resistance [68]		Decreased pulmonary artery pressure [64]
	Human Cardiosphere-derived cell EVs [73]	Anaesthetised juvenile Yorkshire pigs with pressure-induced RV dysfunction [73]	Intramuscular injections of exosomes into the RV free wall [73]	Right ventricular pressure, systemic pressure (femoral artery or left ventricle), and heart rate [73]	Improved right ventricular function [73]	No effect on heart rate, right ventricle pressure, and systemic pressures [73]	
	Human PBMC (known as regeneration-associated cells) and bone marrow-derived MSCs [80]	Conscious Lewis rats with ischaemia-induced injury [80]	Tail vein injection at 30 min, day 1 and day 3 post ischaemia procedure [80]	Ejection fraction [80]	Improved ejection fraction [80]		
Neurological • Traumatic brain injury • Stroke	Endogenous microparticles [61] Endothelial progenitor cells from C57BL/6 mice bone marrow [62]	Anaesthetised new-born pigs [61] Anaesthetised diabetic type II mice subjected to ischaemic stroke [62]	Endogenous EVs released after TBI [61] Tail vein injection two h post ischaemic stroke [62]	Cerebral arteries [61, 62]	Increased cerebral blood flow [62]		Decreased vasodilation [61]
Cirrhosis	Cirrhosis and healthy patients’ platelet-free plasma microparticles [56]	Anaesthetised C57Bl/6 and BALB/C nude mice [56]	Retro-orbital injection 2 h before experiment [56]	Peripheral arterial pressure [56]			Decreased mean [56]
Renovascular disease • Renal artery stenosis	Porcine abdominal adipose tissue MSC-EVs [69] Pig abdominal subcutaneous adipose tissue [70]	Anaesthetised domestic pig model with renal artery stenosis [69] Conscious farm pigs with renal artery stenosis in metabolic syndrome model[70]	Single infusion of EVs into the stenotic kidney [69, 70]	Systolic, diastolic and mean arterial pressure. Renal blood flow and glomerular filtration rate [69]	Increased renal blood flow and glomerular filtration rate [69, 70]	Systolic, diastolic and mean arterial pressure remained elevated [69]	
	Rat white adipose tissue MSC-MVs [79]	Conscious Wistar rats with renal artery stenosis [79]	Tail vein injections at week 3 and 5 post stenosis procedure [79]	Systolic blood pressure [79]			Slight reduction in systolic blood pressure [79]
Pulmonary Hypertension	Human umbilical chord mesenchymal stem cells [67]	Anaesthetised Wistar rats [67]	Daily tail vein injections for 3 days [67]	Right ventricular systolic pressure, heart rate and systolic blood pressure [67]		No difference in heart rate and systolic blood pressure [67]	Decreased right ventricular systolic pressure [67]

#### Effects of EVs studied in anaesthetised animals

A study of traumatic brain injury in a new-born pig model demonstrated impaired pial artery dilation in response to hypotension after traumatic brain injury leading to injury of hippocampal neurons [61]. The authors did not introduce exogenous microparticles of external origin to the animal for this study, instead they aimed to observe the effects of the microparticles that are released by the neurons following the injury, referred to as endogenous microparticles. The authors suggest endogenous microparticles released after injury impaired the vessels’ ability to dilate but it was unclear what the source and cargo of these microparticles [61]. The uncertainty of the source and cargo of the endogenous microparticles is due to a baseline level of circulating microparticles in the animals and the effects observed could be the result of the release of modulators from neurons. In a study using an injured placenta model for preeclampsia demonstrated tail vein injection of placental EVs caused a reduction in cerebral blood flow in a dose-dependent manner in mice, measured non-invasively using laser speckle contrast analysis (LASCA) [42]. The authors demonstrated a similar effect with plasma-derived EVs from preeclamptic patients [42]. In a model of diabetic ischaemic stroke, mice were injected via tail vein with exosomes derived from allogenic endothelial progenitor cells [62]. These injected endothelial progenitor cell-derived exosomes targeted endothelial cells, neurons, microglia and astrocytes in the peri-infarct area, improving cerebral blood flow to this potentially salvageable tissue, leading to a reduction in the infarct size [62]. This effect on cerebral blood flow was increased when the exosomes were enriched with miR126 [62]. Together these results suggest EVs can play a role in the modulation of cerebral blood flow in brain injury.

Four studies investigated the effects of mesenchymal stem cell (MSC)-derived EVs on pulmonary hypertension in rat models [63–67]. Two of the studies sourced the MSCs from Sprague-Dawley rat bone marrow and found the MSC-EVs reduced mean pulmonary arterial pressure and mean right ventricle pressure [63, 64]. Additionally, Chen and colleagues found a decrease in mean arterial pressure [63]. The two studies had differing EV administration protocols, as Chen and colleagues injected EVs intravenously every day for two weeks leading up to the experiments. In contrast, Liu and colleagues injected EVs intravenously once every two days for 35 days, beginning three weeks after induction of pulmonary hypertension by monocrotaline. A subsequent study also found that MSC-EVs injected intravenously before and after induction of pulmonary hypertension normalised right ventricular pressure, and this effect was consistent over a range of doses and dosing intervals [65]. The authors demonstrated a reduction in right ventricle systolic pressure when MSC-EVs were injected into the tail-vein once a day for three days [65], once a week for five weeks or once a fortnight for 10 weeks [66]. These finding suggest MSC-EVs are able to attenuate right ventricular systolic pressure in pulmonary hypertension across the different duration and administration protocols tested across the three studies. MSC-derived exosomes from human umbilical cord MSCs also significantly lowered right ventricular systolic pressure in a rat model for pulmonary hypertension [67]. No differences in heart rate were detected between control and exosome-treated groups [67]. In contrast, in cultured pulmonary arterial endothelial cells transfected to over-express miR-211 increased the mean pulmonary arterial pressure in Sprague-Dawley rats suggesting the involvement of miRNAs in the regulation of the arterial pressure in the pulmonary circuit [68]. Taken together, these papers demonstrate that in models of pulmonary hypertension, MSC-derived-EVs are able to reduce arterial pressure in the pulmonary circulation leading to downstream decreases in right ventricular pressure.

The effects of autologous MSC-EVs on renovascular disease was investigated using a porcine model for renal artery stenosis [69]. The MSC-EVs were infused into the stenotic kidney and four weeks later, single-kidney haemodynamic and function were assessed using multi-detector computed tomography and blood pressure assessed via an intra-arterial catheter [69]. Ferguson and colleagues found the systolic, diastolic and mean arterial pressure remained elevated in the EV-treated stenotic pigs [69]. However, the EV-treated group had an increased renal blood flow and glomerular filtration rate when compared to the control group [69]. In a similar model of kidney stenosis in metabolic syndrome pigs, EVs derived from lean pigs were found to significantly increase renal blood flow and glomerular filtration rate when compared to EVs from metabolic syndrome pigs [70]. These changes are thought to involve inflammatory processes with the authors demonstrating a concurrent increase in renal TGF-β expression, increased regulatory T-cell numbers and a shift in balance towards increased anti-inflammatory M2 and lower pro-inflammatory M1 macrophage phenotypes detected [70].

In the systemic circulation EVs have been demonstrated to have widespread effects. For example, platelet-free plasma EVs from cirrhosis and septic patients decreased mean arterial blood pressure in rodent models [56, 71]. In a different study, both platelet-free plasma EVs from patients with the JAK2 ^V61F^ mutation and erythrocyte microparticles from a mice model with JAK2 ^V61F^ mutation induced an increased response to the vasoconstrictor phenylephrine in the femoral arteries of wild-type mice assessed in vivo [60]. Cardiac function was also assessed using an angiotensin II receptor type 1 (AT1R) knockout mouse model injected intravenously with exosomes from human embryonic kidney cells overexpressing the AT1R [72]. These exosomes expressing AT1R targeted myocytes and mesenteric resistance vessels restoring the responsiveness to angiotensin II typically absent in the AT1R mouse model [72] demonstrating the ability of exosomes to both transfer receptors to target cells, and for these receptors to be functional, affecting the control of cardiovascular function in the anaesthetised mice [72].

The intravenous circulation of EVs can also have localised effects on the systemic circulation. Infusion of erythrocyte-derived EVs resulted in immediate renal vaso-occlusion with a ~ 30% reduction in renal perfusion and vascular congestion of the renal medulla in the kidneys of SAD mice (a model of sickle cell disease), but without effect on systemic arterial blood pressure [27]. Human cardiosphere-derived cell (CDC) exosomes were evaluated in a right ventricular (RV) pressure overload model by banding (constriction) of the pulmonary artery [73]. These exosomes were intramuscularly administered in the free wall of the RV of anaesthetised pigs, and the heart rate, RV pressure and systemic pressures measured via invasive catheters at baseline, and after 28 days [73]. Bittle and colleagues initially did not observe significant changes in the RV-systemic systolic pressure ratio between the treated and control groups. However, by the completion of the study, RV function in the control group remained diminished. Whereas, the RV function of pigs treated with cardiosphere-derived exosomes completely recovered or recovered to 60-70% of baseline depending on the exosome isolation method [73]. The authors used different ultrafiltration and diafiltration methods. The authors demonstrated cardiosphere-derived cell exosomes appear to improve the function of RV when administered intramuscularly.

#### Effects of EVs studied in conscious animals

Four articles using conscious animals found an increase in blood pressure in wild-type animals in response to intravenous injections of EVs from hypertensive models [37, 40, 42, 74]. EVs from miR-27a (novel modulator of hypertension) transfected THP-1 cells demonstrated that miR-27a carrying EVs increased rat systolic blood pressure, and this effect was exaggerated by EVs from lipopolysaccharide-treated THP-1 cells [40]. Two studies using the spontaneous hypertensive rat model found plasma derived-exosomes [37] or EVs [74] from hypertensive rats increase systolic blood pressure. Interestingly, in these two studies, EVs from wild-type rats decreased blood pressure in SHRs [37, 74]. However, administration human platelet microparticles (PMP) alone from the blood of normotensive pregnant women did not significantly improve the systolic blood pressure in a L-NAME induced pregnant rat model for preeclampsia suggesting that it may not be the platelet derived- EVs in plasma that elicited this protective effect [75]. Human MSC-EVs were also demonstrated to maintain systolic and diastolic blood pressure within normal ranges in the DOCA-salt hypertensive rats destined to develop hypertension [76]. The mechanism by which these EVs prevents the development DOCA-salt hypertensive phenotype unclear but may involve the downregulation of inflammatory processes in the kidney [76]. These findings suggest the EVs in hypertensive conditions contains regulatory factors, potentially miRNAs, which delivered intravenously can induce hypertension in normotensive animals. Whereas, introducing EVs from normotensive animals into hypertensive animals has the potential to prevent the development, or reduce the severity of hypertension.

Using the injured placenta model of preeclampsia, infusion of EVs from homogenised mouse placentae into non-pregnant and pregnant mice resulted in the development of hypertension measured by a non-invasive tail-cuff [42]. Similarly, small EVs extruded from extravillous trophoblast cell lines cultured in hypoxic conditions significantly increased mean arterial, systolic and diastolic, blood pressure in pregnant rats [77]. The small-EVs were administered via intravenous injection once a day for four days, and the mean blood pressure was continuously monitored using telemetry catheters inserted in the femoral arteries of the dams [77]. The generation of EVs under hypoxic conditions or released by injured placentae mimic the effects of those generated by plasma EVs in hypertensive models, but whether the underlying mechanisms by which the increased arterial pressure are the same across these models is unclear.

Microparticles modified to carry sonic hedgehog from human lymphoid CEM T-cells were demonstrated to decrease systolic blood pressure in angiotensin II or sodium-induced hypertensive mice [39]. This effect suggests that the cargo of EVs has functional consequences and can influence blood pressure. In contrast, platelet-free plasma microparticles from Crohn’s Disease patients did not decrease blood pressure in mice [78], and MSC-exosomes did not significantly reduce systolic blood pressure in a rat model of renal artery stenosis [79]. These findings suggesting other in vivo factors may mask the effects of these EVs.

The effects of EVs from regeneration-associated cells (RACs) and MSC-derived EVs (MSC-EVs) were assessed in a myocardial ischaemia-reperfusion injury model in conscious rats [80]. After the ischaemia procedure, the rats were administered RAC-EVs or MSC-EVs via tail vein injection. The injections were repeated on day 1 and day 3 post-operation [80]. After four weeks, the ejection fraction was significantly improved in the RAC-EV group compared to the MSC-EV and control groups with the authors suggesting RAC-EVs were more effective at preserving the ejection fraction than MSC-EVs post myocardial ischaemia [80].

### Summary of data

**Fig. 2:**
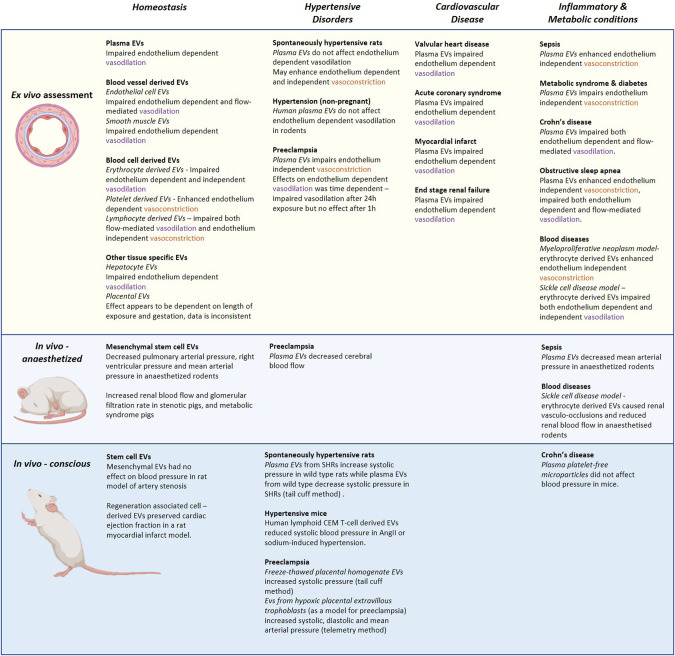
Summary of the effects of extracellular vesicles on vascular haemodynamics under homoeostatic and disease states

In summary, the current literature suggests that in non-pathologic models, EVs of various origins impair endothelium-dependent vasodilation ex vivo [2] (Fig. 2). Impaired endothelium-dependent vasodilation has been observed when studying the effects of a heterogenous mix of plasma EVs, isolated endothelial cell derived EVs, erythrocyte and lymphocyte-derived EVs. In contrast, this effect is absent in both human hypertension and the SHR model. Plasma EVs were studied predominantly in pathologies, where cardiac diseases, sickle cell anaemia, Crohn’s disease, obstructive sleep apnoea all were reported to impair endothelium-dependent vasodilation ex vivo. In contrast, the effect of EVs on vasoconstriction is variable and dependent on the pathology studied. Fewer studies were performed in vivo and findings from these publications, was not always consistent with data from corresponding models ex vivo.

## Discussion

Evidence presented in this systematic review supports the notion that EVs can affect cardiovascular haemodynamics under both pathologic and non-pathologic conditions. Under homoeostatic conditions, EVs from all origins studied (plasma-derived, blood vessel, blood cells, hepatocyte and placental trophoblast-derived) impaired endothelium-dependent vasodilation, as well as flow-mediated vasodilation. Endothelium-independent vasodilation (i.e. via sodium nitroprusside) was less affected. Taken together, this suggests that EVs may potentially play a role in the modulation of normal vascular tone, primarily via their effects on endothelial cells. Few studies found an effect of EVs on reactivity to vasoconstrictors ex vivo. However, of the studies which found an effect, whether this enhanced or impaired reactivity to the vasoconstrictors tested was dependent on the origin of the EVs. Whilst we expect that these differences may contribute to changes in vascular haemodynamics, this is very hard to test in vivo given the large number of endogenous EVs of differing cellular origins in all model organisms. Three studies injected MSC-EVs to test their effects in vivo. Their findings were that MSC-EVs appeared to decrease pulmonary, ventricular and arterial pressures [63–66], which is in contrast to the ex vivo wire and pressure myography data on EVs from other cells under homoeostatic conditions. However, this difference is consistent with the known immunomodulatory and therapeutic properties of MSCs and their EVs.

As expected, the effect of human EVs from pathologic conditions and in animal models of pathology, appeared to be largely dependent on the nature of the pathology and the pathological/health/physiological status of the recipient vessels. Interestingly, plasma EVs from non-pregnant hypertensive patients and SHR rats did not affect endothelium-dependent vasodilation. In contrast, in both homoeostasis and in cardiac and renal disease, plasma EVs impaired endothelium-dependent vasodilation, confirming that in hypertension, the source of the EVs and/or the function of the cargoes in the EVs is altered. It has been demonstrated that in hypertension, the proportion of plasma EVs originating from endothelial cells is increased [81–83] and changes in the miRNA profiles are also altered during pulmonary hypertension [84]. It has previously been suggested that the different effects on vasodilation of plasma EVs in hypertension could be a compensatory response to the vasoconstrictive state of hypertension [10]. Furthermore, it also appears that the effects of plasma EVs from hypertensive disorders are dependent on the pregnancy status of the affected individual. In preeclampsia, plasma EVs were shown to impair endothelium-dependent vasodilation, despite the being a hypertensive disorder. In very early pregnancy, there are changes in the maternal physiology including an increase in the heart rate and stroke volume, as well as, total blood volume [85]. To prevent the pregnant individual from developing hypertension there is a compensatory decrease in peripheral vascular resistance. Preeclampsia is thought to arise when the compensatory decrease in vascular resistance is reduced or absent. There is growing evidence that EVs contribute substantially to these maternal cardiovascular adaptations in normal pregnancy [86] and that alterations in the composition of EVs contribute to the development of hypertension and preeclampsia. The discrepancy in the effects of plasma EVs in hypertension vs preeclampsia may be due to additional compensatory mechanisms in the pregnant state [45]. Alternatively, there is a new source of EVs in pregnant women; the placenta. The placenta continuously releases large numbers of EVs into the maternal circulation each day with placental EVs contributing 10% of EVs in the maternal circulation [36]. The literature suggests the miRNA and protein cargoes of these placental EVs are also altered in preeclampsia [87–89], however, a direct comparison of the effects of placental derived EVs on vascular reactivity from women with preeclampsia and normotensive pregnant women is lacking.

### Extracellular vesicles are a heterogenous and experimentally loosely classified by their size range and in some cases biogenic origins

Extracellular vesicles are heterogenous in nature, can be a range of sizes and may contain different cargoes depending on the cell of origin [90]. As the field of EV research is still quite novel, guidance for the nomenclature of these particles is described in the ‘Minimal Information for studies of extracellular vesicles (MISEV) 2018’ guideline [3]. This guideline suggests that EVs should be categorized by their size, biochemical composition, or description of cells/cellular origin.

Methods of isolation of EVs also varied in the studies included in this systematic review. This is reflective of the state of the current field of EVs where there are multiple accepted isolation methods, each with their strengths and draw backs. For example, the majority of studies reviewed employed ultracentrifugation as a method of isolation. It is known that EV samples isolated by ultracentrifugation may be contaminated by free proteins and protein aggregates, whilst microvesicle samples isolated using this method may be contaminated by smaller EVs (including exosomes) [91]. Both these contaminants have a potential to influence the outcome of the experiments leading to confounding of whether the effects observed were because of the EVs (and the cargo it carries), or due to the free proteins contaminating the preparation. Furthermore, there are contaminating non-EV lipid particles such as high-density lipoproteins and very low-density lipoproteins that can also be isolated with various isolation methods [92]. Whilst we do not know whether the isolation methodology alters the effect of the EVs on vascular haemodynamics, it has been previously demonstrated that differences in isolation methodology can itself change the biodistribution of the EVs [93]. The isolation methods for EVs and characterization of the EVs as per the MISEV 2018 guidelines reported in the included articles in this review are highlighted in Supplementary Table 1. In this review, the majority of the papers reviewed employed ultracentrifugation as the isolation technique which does not demonstrate that the injected samples were a pure population of EVs. It is highly likely that the observed outcomes is at least in part due to the contaminant collected during the isolation process, such as lipoproteins or free protein/protein aggregates and not entirely attributable to the effect of EVs themselves.

### Techniques employed were variable

A further major variation identified between the studies included in this review was dose and time of exposures. EVs were delivered variously via extravascular incubation in the myograph bath, intraluminal incubation in isolated vessel, single intravenous injections in live animals or multiple injections over weeks. Tong and colleagues demonstrated that the effects of placental EVs under homoeostatic conditions were different between 30 min and 24 h exposure times in vivo [34]. Similarly, Van Wijk and colleagues also demonstrated a time-dependent response to placental microvesicles [41]. It is likely that time and dose-dependent effects exist that may not be captured in many of the reviewed studies that in part account for the differences seen between studies.

There was further variation in the vasodilator drugs tested in the manuscripts reviewed with some studies using acetylcholine (endothelial cell dependent) while others used sodium nitroprusside (endothelial cell independent), angiotensin 1–7 or bradykinin. There are multiple mechanisms that lead to vasodilation and vasoconstriction in arterial vessels. In this review, we have loosely classified vasodilation into endothelium-dependent and endothelium-independent but acknowledge that some of these mechanisms overlap. For example, while acetylcholine is an endothelium-dependent vasodilator, it shares downstream pathways to sodium nitroprusside, which we have classified as endothelium-independent. Additionally, enhanced reactivity to vasoconstrictors such as angiotensin II, phenylephrine or serotonin may occur through different mechanisms and altered reactivity to one vasoconstrictor does not necessitate altered reactivity to another. In order to understand the higher-level picture of how EVs affect the cardiovascular haemodynamics, we have ignored the different mechanisms of action and generalised to increased or decreased response to vasodilators or vasoconstrictors.

### Lack of randomisation and blinding in animal studies

Research articles included in this review use a variety of approaches to assess different aspects of vascular function both ex vivo and in vivo. Some approaches, such as non-invasive blood pressure measurements are more prone to increased risk of bias during analysis unless performed and analysed with the researcher blinded. However, the vast majority of articles included in this review did not report blinding, with the exception of Mortaza et al. [71], Poisson et al. [60], and Ren et al. [74] (detailed in Supplementary Table 1). Furthermore, in most animal studies, there were no details on the randomization of treatment with EVs versus control thus increasing the potential for confounding.

### Concluding summary

In this review, we have used derived from the PRISMA guidelines to systematically reviewed the literature for studies which assessed the effect of extracellular vesicles on vascular haemodynamics. Here, we have identified that with the exception of hypertensive disorders, EVs appear to impair endothelium-dependent vasodilation in both homoeostasis and pathology, including cardiovascular-renal diseases. The effects of EVs on vasoconstriction are more variable, and are, as might be expected, dependent on pathology studied and the cellular origin of the EVs. The findings of this review suggest that EVs, are involved in the modulation of vascular tone under both normal and pathologic conditions.

Robust, repeatable and comparable studies are essential to further our understanding of the role of EVs in the cardiovascular system, including understanding the mechanism by which these effects may occur, and which cargo may be responsible. As this topic intersects the fields of EV biology and cardiovascular physiology, it is essential that suggested guidelines across both fields are met and reported in the publication to increase confidence in the published research.

### Supplementary information

Supplementary information
