# Supplementary material for: Extracellular vesicles and their effect on vascular haemodynamics: a systematic review
Source: Hypertens Res. 2024 Apr 10;47(6):1588–606. doi: 10.1038/s41440-024-01659-x (PMC11150158; doi:10.1038/s41440-024-01659-x)
Supplement: Supplementary file 1 — Supplementary information [file 41440_2024_1659_MOESM1_ESM.docx]

**Supplementary Table 1: ARRIVE and MISEV guideline aspects reported in the publications**

| **Article** | **Aspects of ARRIVE Guidelines** | | **Aspects of MISEV Guideline (2018)** | |
| --- | --- | --- | --- | --- |
|  | **Randomisation** | **Blinding** | **EV Isolation Method** | **Characterisation of EVs**  i. At least three positive protein markers of EVs, including at least one  -transmembrane/lipid-bound protein  -cytosolic protein  ii. At least one negative protein marker |
| Agouni et al. 2007 (50) | Randomisation method not stated.  Mice were treated with an IV injection of either MPs, or the same volume of vehicle in the absence of presence of cyclopamine. | Experiments were not blinded. | Centrifugation   - 750g - 1,500g - 14,000g for three steps | Characterisation was not stated. |
| Agouni et al. 2008 (57) | Randomisation method not stated.  Mice were treated with an IV injection of circulating levels of MPs of either Metabolic Syndrome patients or healthy subjects. | Experiments were not blinded. | Centrifugation   - 270g - 1,500g - 21,000g for three spins | Flow cytometry for antigen expression on circulating MPs:  Endothelial MPs – anti-CD146.  Platelet, erythrocyte, and leukocyte – antiCD41, anti-CD235a, and anti-CD45.  P-selectin+ and L-selectin+ MPs – anti 62L and anti-CD62P.  Phosphatidylserine-expressing MPs – Annexin V. |
| Agouni et al. 2011 (55) | Randomisation method not stated.  Mice were treated with an IV injection of either vehicle medium or MPs at the circulating level detected in each health subject or Metabolic Syndrome patient. | Experiments were not blinded. | Centrifugation   - 270g for 20 minutes - 1,500g for 20 minutes - 21,000g for 45 minutes - Two spins at 14,000g for 45 minutes | Flow cytometry to quantify circulating MP levels.   - Platelet population – CD41+ - Endothelial population – CD146+ - Erythrocyte population – CD235+ - Pro-coagulation - annexin V+ |
| Amabile et al. 2005 (51) | Randomisation method not stated.  Rat thoracic aortas were incubated in the presence of MPs from end-stage renal failure patients (circulating levels), or MPs from health subjects (at circulating levels of ESRF patients) or MP supernatant. | Flow cytometry characterisation was blinded, examiners were unaware of the subject status. | Centrifugation   - Platelet-free plasma was separated from whole blood - 20,500g for 45 minutes - Pellet was treated with DMEM supplemented with streptomycin, penicillin and polymyxin B. - 20,500g for 45 minutes   Magnetic pan-mouse IgG Dynabeads were used to remove platelet-, erythrocyte-, and leukocyte-derived MPs. The remaining MP suspension was further centrifuged at 20,500g for minutes. | Flow cytometry to define specific MP populations:   - Anti-CD31-phy-coerythrin - Anti-CD41-PC5 - Anti-CD144-PE - Anti-CD235a-FITC - Anti-CD3-FITC - Anti-CD11b-PC5 - Anti-CD45-FITC - Anti-CD66b-FITC - Phosphatidylserine MPs – fluorescein-conjugated Annexin V   Defined MPs as elements between <1µm and >0.1µm in size and were positively labelled by specific antibodies above. |
| Bittle et al. 2020 (73) | Randomisation method not stated. | Experiments were not blinded. | Ultrafiltration via centrifugation   - Initial 10kDa molecular cut-off weight to concentrate the EVs - Sample XO-2 differed from XO-1 by 1000kDa - Sample XO-3 differed from XO-2 by 10kDa | Protein quantified, particle quantified, and mean particle size described. |
| Bohman et al. 2016 (61) | Randomisation method not stated. | Experiments were not blinded. | Centrifugation   - Aliquots of blood were centrifuge at 2,300g for 15 minutes | Flow cytometry to quantify MPs   - Annexin V+ |
| Boisrame-Helms et al. 2015 (17) | Randomisation method not stated. | Experiments were not blinded. | Centrifugation   - 1,500g for 15 minutes - 13,000g for 2 minutes - 13,000g for 45 minutes   MPs were washed with TBS containing 3% w/v human serum albumin by double-step centrifugation at 12,000g for 60 minutes. | Pro-thrombinase assay to measure insoluble MPs |
| Boulanger et al. 2001 (46) | Randomisation method not stated. | Experiments were not blinded. | Centrifugation   - 11,000g for 2 minutes - 13,000g for 45 minutes | Protein content measured by Biorad assay. |
| Brodsky et al. 2004 (21) | Randomisation method not stated. | Experiments were not blinded. | Centrifugation   - 5,000g for 10 minutes - 100,000g for 2 hours | Flow cytometry to measure quantity of MPs   - Defined as CD31+/CD42- or CD51+ - Size greater than 1.5µm |
| Burger et al. 2016 (22) | Randomisation method not stated. | Experiments were not blinded. | Centrifugation   - 2,500g for 10 minutes - 20,000g for 20 minutes | Flow cytometry to confirm and quantify MPs   - Annexin V |
| Camus et al. 2012 (27) | Randomisation method not stated. | Experiments were not blinded. | Centrifugation   - 400g for 15 minutes - 12,500g for 5 minutes – twice | Flow cytometry was used to characterise the MPs   - Annexin V - Anti-Ter119 antibody   Flowcount used to quantify MPs   - Defined as 0.1 to 1µm in diameter |
| Chen et al. 2014 (63) | Rats were randomly assigned to either vehicle, MSC or MSC-EVs. | Experiments were not blinded. | Centrifugation   - 2,000g for 20 minutes - 100,000g for 1 hour | Bradford method used to measure protein concentration.  Flow cytometry   - CD29 - Annexin V - IgG nonspecific antibody as negative control   Transmission electron microscopy – experiments were conducted in triplicate.  Nanoparticle tracking analysis used to determine size of EVs. |
| Densmore et al. 2006 (24) | Randomisation method not stated. | Experiments were not blinded. | Centrifugation   - 300g for 10 minutes - 100,000g for 60 minutes | Flow cytometry used to characterise MPs, anti-human antibodies used:   - Tissue factor - E-selectin - CD31 - Isotype control   Flow cytometry also used to determine concentration and size of MPs for each sample. |
| Dutta et al. 2020 (77) | Dams were randomly divided into treatment and control group. | Experiments were not blinded. | Centrifugation   - 2,000g for 10 minutes - 2,000g for 15 minutes   Supernatant was filtered then subjected to size-exclusion chromatography. | Nanoparticle tracking analysis used to determine EV concentration.  Electron microscopy used to determine morphology.  Western blot analysis to determine protein associated with the EVs. |
| Essayagh et al. 2005 (26) | Randomisation method not stated. | Experiments were not blinded. | Centrifugation   - Low speed centrifugation - 12,500g for 1 hour | Flow cytometry used to quantify MPs and exposure of surface molecules.   - Annexin V - Anti-mouse CD61 - Anti-human CD142 - Isotype control |
| Fang et al. 2020 (75) | Normotensive women that gave birth at term with randomly selected as the control group.  Rats were randomly divided into the treatment combination and control groups. | Experiments were not blinded. | Centrifuged to obtain platelet-free plasma. | Fluorescence-activated cell sorting (FACS)   - 0.1µm, 0.3 µm, 0.5 µm and 0.9 µm beads used.   Platelet-microparticles defined as CD41 and Annexin V positive.  IgG non-specific used as control. |
| Ferguson et al. 2021 (69) | Animals were randomised into diseased-state or control groups.  Diseased-state animals were randomised to receive EVs, fat tissue collection, harvest of autologous mesenchymal stem cells and their daughter EVs. | Experiments were not blinded. | Ultracentrifugation | EVs were characterised by:   - CD63+ - CD9+ - CD81+ |
| Freed et al. 2017 (25) | Randomisation method not stated. | Experiments were not blinded. | Centrifugation   - 140g for 8 minutes - 100,000g for 1 hour | Flow cytometry used to quantify EVs   - Annexin V+ and CD31+ were defined as EVs   Liquid chromatography tandem mass spectrometry used to detect protein composition. |
| Fu et al. 2015 (48) | Randomisation method not stated. | Experiments were not blinded. | Centrifugation   - 11,000g for 2 minutes - 13,000g for 45 minutes | Flow cytometry   - Annexin V+ and within 1µm |
| Gaceb et al. 2016 (54) | Randomisation method not stated. | Experiments were not blinded. | Centrifugation   - Centrifuged to obtain PFP - 21,000g for 45 minutes | Malvern Zetasizer to quantify EVs.  Flow cytometry to identify progenitor of EVs   - CD61 - TER119 - Anti-CD45 - Anti-CD54 - Anti-CD133 - Scal-PC7 - IgG as a negative control |
| Ge et al. 2021 (67) | Animals were randomly divided into four groups: control, disease model, disease model with exosomes, and disease model with culture medium. | Experiments were not blinded. | Centrifugation   - 3,000g for 10 minutes - 2,000g for 10 minutes - 10,000g for 30 minutes - 100,000g for 1 hour | Transmission electron microscopy used to identify and observe morphology of EVs.  Flow cytometry.  Bicinchoninic acid assay and western blot used to quantify protein of EVs. |
| Good et al. 2020 (10) | Randomisation method not stated. | Experiments were not blinded. | Centrifugation   - 3,250g for 10 minutes - 17,000g or 20,000g for 30 minutes - 116,000g for 75 minutes | Nanoparticle tracking analysis used to detect size of EVs.  Flow cytometry used to detect protein content   - CD105 - CD31 - Annexin V - CD45 - CD42 |
| Han et al. 2015 (49) | Randomisation method not stated. | Experiments were not blinded. | Centrifugation   - 11,000g for 2 minutes - 13,000g for 45 minutes | Bicinchoninic acid assay used to quantify protein content.  Flow cytometry was used to identify progenitors of EVs   - CD31 - CD41 - CD14 |
| Han et al. 2020 (42) | Randomisation method not stated. | Experiments were not blinded. | Centrifugation   - 1,500g for 20 minutes - 13,000g for 5 minutes - 100,000g for 1 hour twice | Flow cytometry used to measure levels of EVs   - Placental alkaline phosphatase - Syncytin |
| Ishida et al. 2016 (58) | Randomisation method not stated. | Experiments were not blinded. | Centrifugation   - 1,900g for 10 minutes - 3,000g for 10 minutes - 15,000g for 45 minutes | Dot blotting characterisation   - CD61 - CD62P   Flow cytometry   - Annexin V   ELISA – platelet-derived microparticles kit used. |
| Ishiy et al. 2020 (79) | Randomisation method not stated. | Experiments were not blinded. | Ultracentrifugation   - 3,000g for 20 minutes - 10,000g for 30 minutes - 100,000 for 2 hours | Nanosight to identify EVs via particle analysis.  Western blot   - MMP2 for microveiscles - CD63 for exosomes |
| Klinger et al. 2020 (65) | Randomisation method not stated. | Experiments were not blinded. | Centrifugation   - 300g for 10 minutes - 100,000g for 1 hour | Nanoparticle tracking analysis used to quantify EVs.  Bicinchoninic acid assay used to quantify protein content.  Electron microscopy and Western blot used to characterise EVs. |
| Klinger et al. 2021 (66) | Randomisation method not stated. | Experiments were not blinded. | Centrifugation   - 300g for 10 minutes - 100,000g for 1 hour | Bicinchoninic acid assay used to quantify protein content.  Electron microscopy and particle size distribution were analysed. |
| Leonetti et al. 2013 (78) | Randomisation method not stated. | Experiments were not blinded. | Centrifugation   - 270g for 20 minutes - 1,500g - 21,000g for 90 minutes | MP subpopulations and concentration were characterised by flow cytometry   - CD41 - CD235 - CD45 - CD146 - CD66b - CD62P - CD62L - IgG was a isotype matched negative control |
| Lindoso et al. 2020 (76) | Animals were randomly divided into three cohorts. | Experiments were not blinded. | Centrifugation   - 2,000g for 20 minutes - 100,000g for 2 hours | Nanoparticle tracking analysis used to characterise size and quantification. Repeated three times per sample.  Flow cytometry used to qualitatively analyse surface markers.   - CD62 - CD9 - CD81 - Secondary antibodies was a negative control   Transmission electron microscopy used to observe morphology. |
| Liu et al. 2018 (64) | Animals were randomly divided into control, diseased-state or treatment group. | Experiments were not blinded. | Centrifugation   - 2,000g for 20 minutes - 100,000g for 1 hour twice | Transmission electron microscopy used to observe morphology.  Bicinchoninic acid assay used to quantify protein concentration. |
| Mahmoud et al. 2017 (23) | Randomisation method not stated. | Experiments were not blinded. | Centrifugation   - 4,300g for 5 minutes - 100,000g for 2 hours | Flow cytometry used to quantify EVs   - Annexin V   Bicinchoninic acid assay used to quantify protein concentration.  Western blot used to identify proteins. |
| Marrachelli et al. 2013 (39) | Randomisation method not stated. | Experiments were not blinded. | Centrifugation   - 750g for 15 minutes - 1,500g for 5 minutes - 14,000g for 45 minutes | Braford method with BSA was used to determine amount of EVs. |
| Martin et al. 2004 (28) | Randomisation method not stated. | Experiments were not blinded. | EVs from CEM T cells were isolated via 4 centrifugation steps.  EVs from patients were isolated via 2 step centrifugations. | Microtitration plates were used to capture EVs via immobilised annexin V. |
| Meziani et al. 2006 (18) | Randomisation method not stated. | Experiments were not blinded. | Centrifugation   - 1,500g for 15 minutes - 14,000g for 2 minutes - 13,000g for 45 minutes | Flow cytometry used to determine phenotype of EVs.   - GPIbα - CD11a - CD31 |
| Murugesan et al. 2022 (32) | Randomisation method not stated. | Experiments were not blinded. | Centrifugation   - 3,000g for 20 minutes - 1,500g for 20 minutes - Filtered through a 0.22µm | Transmission electron microscopy used to analyse size and morphology.  Nanoparticle tracking analysis used to determine the size distribution.  Bicinchoninic acid assay used to quantify protein concentration.  Immune blotting   - CD63 - Flotilin-1 |
| Mortaza et al. 2009 (71) | Animals were randomly allocated into control, sham and treatment groups. | Operators were blinded for the administration of EVs to animals. | Centrifugation   - 170g for 10 minutes - 1,500g for 20 minutes - 21,000g for 45 minutes twice | Flow cytometry was used to phenotype the EVs   - Anti-CD61 - Anti-CD45 - Anti-CD54 - Annexin V |
| Mostefai et al. 2008 (52) | Randomisation method not stated. | Experiments were not blinded. | Centrifugation   - 1,500g for 15minutes - 14,000g for 2 minutes - 14,000g for 45 minutes | Flow cytometry used to determine EV subpopulations   - CD146+ - CD62L+ - CD62P+ - CD45+   Western blot   - eNOS - caveolin-1 - iNOS - NF-κB - Nox-1 - Nox-4 - P-i κBα - MnSOD |
| Otani et al. 2018 (37) | Randomisation method not stated. | Experiments were not blinded. | Ultracentrifugation   - 1,500g for 10 minutes - 9,481g for 30 minutes - 164,071g for 35 minutes | Size distribution was measured by qNANO with a NP100 nanopore at 46.5mm. |
| Pfister et al. 2004 (29) | Randomisation method not stated. | Experiments were not blinded. | Centrifugation   - 1,500g for 10 minutes - 1,500 for 15 minutes - 13,000g for 30 minutes | Characterisation not described. |
| Pironti et al. 2015 (72) | Randomisation method not stated. | Experiments were not blinded. | Centrifugation   - 1,000g for 15 minutes - 12,000g for 20 minutes - 18,000-20,000g for 20 minutes - Filtered through 0.22µm filter - 100,000g for 70 minutes | Nanoparticle tracking analysis used to determine size distribution.  Transmission electron microscopy used to analyse morphology of EVs.  Immune blotting   - CD9 - CD63 - Alix - Calnexin |
| Poisson et al. 2020 (60) | No randomisation was used in these experiments. | Investigators were not blinded to group allocation during collection and analysis data. | Centrifugation   - Human samples = 2,500g for 15 minutes twice then 20,500g for 2 hours - Mice samples = 15,000 for 1 minutes and 20,500 for 45 minutes | Flow cytometry used to analyse concentration of EVs   - Annexin V   Size-exclusion chromatography to separate out EVs from proteins.  Western blots and Bicinchoninic acid assay used to identify and quantify protein concentration. |
| Pricci et al. 2009 (30) | Randomisation method not stated. | Experiments were not blinded. | Centrifugation   - 750g for 15 minutes - 1,500f for 5 minutes - 14,000g for 45 minutes | Concentration of protein carried by EVs were quantified by protein assay. |
| Priou et al. 2010 (53) | Randomisation method not stated. | Experiments were not blinded. | Centrifugation   - 270g for 20 minutes - 1,500g for 20 minutes - 21,000g for 45 minutes | Flow cytometry used to identify subpopulations of EVs   - CD41 - CD235a - CD45 - CD146 - CD66b - CD62P - CD63L - Annexin V |
| Rautou et al. 2012 (56) | Randomisation method not stated. | Experiments were not blinded. | Centrifugation   - 500g for 15 minutes - 15,200g for 5 minutes | ELISA used to measure hepatocyte derived EVs.  Flow cytometry used to analyse EVs   - CD11a - CD31 - CD235a - CD14 - CD4 |
| Ren et al. 2019 (74) | Animals were randomly divided into three groups – control, EV treatment, and EV treatment in disease model. | Experiments were double blinded. | Centrifugation and PureExo exosome isolation kit. | Morphology analysed with transmission electron microscopy.  Nanoparticle tracking analysis used to determine size distribution of EVs.  Bicinchoninic acid assay used to quantify protein concentration. |
| Royo et al. 2017 (12) | Animals were randomly allocated into four groups. | Experiments were not blinded. | Centrifugation   - 110,000g overnight - 1,500g for 10 minutes - Filtration on 0.22µm pore - 10,000g for 30 minutes - 100,000g for 75 minutes | Western blot used to measure arginase activity and identify proteins.  Cryo-electron microscopy used to analyse morphology of EVs.  Nanoparticle tracking analysis used to determine size distribution. |
| Salybekov et al. 2021 (80) | Randomisation method not stated. | Experiments were not blinded. | Centrifugation   - 300g for 10 minutes - 2,000g for 20 minutes - Filtration through a 0.2µm filter - 174,000g for 110 minutes | EV number and size was measured using nanoparticle tracking analysis.  Transmission electron microscopy used to characterise morphology of EVs.  Flow cytometry used to identify EV progenitors   - CD9 - CD63 - Alix - Hsp-70 |
| Song et al. 2020 (70) | Animals were randomised to two diet groups. | Experiments were not blinded. | Centrifugation   - 2,000g - 100,000g for 1 hour | Flow cytometry was used to characterise EVs   - CD40 - CD9 - CD81 - Β1 |
| Spaans et al. 2017 (33) | Arterial segments were randomly assigned to experimental groups. | Experiments were not blinded. | Centrifugation   - 150,000g | Flow cytometry used to confirm placental origin of EVs.  Nanoparticle tracking analysis used to determine size distribution. |
| Spaans et al. 2018 (35) | Randomisation method not stated. | Experiments were not blinded. | Centrifugation   - 1,500g microvesicles - 150,000g nanovesicles | Flow cytometry used to confirm placental origin of EVs.  Nanoparticle tracking analysis used to determine size distribution. |
| Tesse et al. 2005 (31) | Randomisation method not stated. | Experiments were not blinded. | Centrifugation from cell cultures   - 750g for 15 minutes - 200g for 5 minutes - 14,000g for 45 minutes twice   Centrifugation for circulating samples   - 1,500g for 15 minutes - 13,000g for 2 minutes - 13,000g for 45 minutes | Prothrombinase assay used to determine MP amount and phenotypes. |
| Tesse et al. 2007 (19) | Incubation of vessel with EVs were randomised. | Experiments were not blinded. | Centrifugation   - 1,500g for 15 minutes - 14,000g for 2 minutes - 13,000g for 45 minutes | Nanomolar phosphatidylserine equivalents used to quantify EVs.  EVs were phenotyped with specific monoclonal antibodies   - GPIβa - CD11a - CD31 |
| Tual-Chalot et al. 2012 (59) | Randomisation method not stated. | Experiments were not blinded. | Centrifugation   - 270g for 20 minutes - 1,500g for 20 minutes - 21,000g for 45 minutes | Flow cytometry used to determine EV subpopulations and concentration   - CD41 - CD45 - CD146 - CD62L - Annexin V |
| VanWijk et al. 2002 (41) | Randomisation method not stated. | Experiments were not blinded. | Centrifugation   - 1,550g for 20 minutes - 17,575g for 30 minutes | Characterisation of EVs not stated. |
| Wang et al. 2020 (62) | Animals were randomly assigned treatment groups. | Experiments were not blinded. | Centrifugation   - 300g for 30 minutes - 2,000g for 20 minutes - 20,000g for 70 minutes - Filtered through a 0.22µm syringe filter - 100,000g for 90 minutes | Nanoparticle tracking analysis used to determine size distribution.  MACS with NTA used to analyse level of endothelial progenitor cell EVs. |
| Ye et al. 2017 (47) | Randomisation method not stated. | Experiments were not blinded. | Centrifugation   - 11,000g for 2 minutes - 13,000g for 45 minutes | Bicinchoninic acid assay used to quantify protein concentration.  Flow cytometry used to determine subpopulations of EVs   - CD31 - CD41 |
| Zhang et al. 2021 (68) | Animals were randomly divided into control or disease-state groups. | Experiments were not blinded. | Centrifugation   - 4,300g for 5 minutes - 14,000g for 35 minutes - 110,000g for 1 or 2 hours | Transmission electron microscopy was used to analyse the morphology of EVs.  qRT-PCR used to detect expressions of miRNA. |
| Zhaorigetu et al. 2020 (38) | Randomisation method not stated. | Experiments were not blinded. | Sequential filtration assay. | Transmission electron microscopy was used to analyse the morphology of EVs.  RNA and protein assay.  Quantification of EVs via qNano. |
| Zou et al. 2020 (40) | Randomisation method not stated. | Experiments were not blinded. | Centrifugation, filtration then ultracentrifugation.   - 500g for 20 minutes - 1,500g for 20 minutes - 110,000g for 70 minutes | Transmission electron microscopy was used to analyse the morphology of EVs.  Flow cytometry used to count EVs.  Immune blotting used to identify proteins in EVs. |
